# No pain, no gain revisited: the impact of positive and negative psychotherapy experiences on treatment outcome

**DOI:** 10.3389/fpsyg.2024.1378456

**Published:** 2024-06-18

**Authors:** Roos Verkooyen, Nick J. Broers, Brechje Dandachi-FitzGerald

**Affiliations:** ^1^METggz, Maastricht, Netherlands; ^2^Zuyderland Medisch Centrum, Geleen, Netherlands; ^3^Department of Clinical Psychological Science, Maastricht University, Maastricht, Netherlands; ^4^Faculty of Psychology, Open University, Heerlen, Netherlands

**Keywords:** psychotherapy outcome, positive psychotherapy experiences, negative psychotherapy experiences, side effects of psychotherapy, adverse events

## Abstract

**Objective:**

Psychotherapy may have many benefits for patients with mental health problems, but patients may also have negative experiences related to the therapy. Not much is known about these negative psychotherapy experiences and their impact on treatment outcome. The aim of this study was to examine the association between positive and negative psychotherapy experiences and treatment outcome.

**Methods:**

A total of 130 patients participated in the study. They received treatment as usual and were assessed for positive and negative psychotherapy experiences at mid-treatment and post-treatment using the Positive and Negative Experiences of Psychotherapy Questionnaire (PNEP). Treatment outcome was measured by the Outcome Questionnaire - 45 (OQ-45). Multiple linear regression was used to analyze the data.

**Results:**

All patients reported positive psychotherapy experiences at mid-treatment. At least one negative experience was reported by 69% of participants. After correction for baseline severity (i.e., OQ-45 at baseline) and relevant demographic variables, positive psychotherapy experiences at mid-treatment emerged as a predictor for treatment outcome. In contrast, negative psychotherapy experiences did not predict treatment outcome. However, reporting more negative experiences was associated with reporting fewer positive experiences at mid- and end of treatment.

**Conclusion:**

Both positive and negative psychotherapy experiences commonly occur. Although in this study negative psychotherapy experiences did not predict treatment outcome in terms of symptom reduction, the findings do suggest that negative experiences may influence the way in which patients evaluate their treatment. Although positive experiences outweigh negative experiences, patients should be informed that negative experiences may also occur.

## Introduction

Over the past few decades, research has evidently shown that psychotherapy is effective in reducing psychological distress and improving quality of life for many patients (Holmes et al., 2018; Wakefield et al., 2020; Cuijpers et al., 2023). However, much less is known about the potential negative effects of psychotherapy. Little attention has been paid to the possibility that not all patients will improve, and some may even deteriorate or have unwanted negative experiences during therapy (Lilienfeld, 2007; Holmes et al., 2018; Rozental et al., 2018).

The extent to which negative effects of psychotherapy exist, and the problems these effects may cause, has been the subject of much debate in the past (Barlow, 2010). Some of the earliest evidence that interventions designed to improve human behavior can also have unintended, harmful consequences, is the Cambridge-Somerville Youth Study (Powers and Witmer, 1951). This longitudinal study was conducted in the 1940s with the aim of reducing juvenile delinquency. As described by McCord (2003), in this study, pre-delinquent boys were randomly assigned to a control condition or a treatment condition in which they received counseling and individualized services. Contrary to the researchers’ expectations, follow-up showed that boys in the treatment group were more likely to have undesirable outcomes, such as involvement in criminal activity, receiving a mental health diagnosis, or even dying at a younger age. In exploring the explanations for these findings, the authors suggested one possible explanation: the summer camps may have provided an opportunity for boys to model and reinforce each other’s problem behaviors. A recent meta-scientific review focusing on potentially harmful therapies (Williams et al., 2021), examining over 70 studies, found evidence that some interventions have the potential to cause harm. Evidence of potential harm was most clearly evident for Scared Straight interventions, which aim to deter future criminal behavior by exposing at-risk youth to the dangers of prison life, and Critical Incident Stress Debriefing.

One of the first systematic analyses of the negative effects of psychotherapy was provided by Bergin (1966), who reviewed seven psychotherapy outcome studies in which no apparent differences were found between treatment and control groups. On closer examination of the data, this author found a wider range of change scores in the treatment groups than in the control groups, with the former showing both greater improvement and greater deterioration on outcome measures after therapy. These findings suggest that psychotherapy not only has the potential to cause some patients to improve in comparison to untreated patients, but also to cause a proportion of patients to deteriorate significantly more. Despite these findings, research in the following decades has continued to focus primarily on the positive effects of psychotherapy, such as symptom reduction and improved well-being. This has provided important evidence for the effectiveness of psychotherapy for many psychiatric disorders, but the potential negative effects of psychotherapy have often been overlooked (Dandachi-Fitz Gerald et al., 2023).

Berk and Parker (2009) argued that costs and benefits are conceptualized differently in pharmacotherapy and psychotherapy. For example, monitoring for negative effects is mandatory in medical research, but not in psychotherapy research (Nutt and Sharpe, 2008). A study by Vaughan et al. (2014) compared 45 randomized controlled trials of pharmacotherapy and psychotherapy (15 pharmacotherapy only trials, 15 psychotherapy only trials, and 15 combined pharmacotherapy and psychotherapy trials) on the frequency of reporting of adverse events, defined as deleterious results directly attributable to a treatment intervention. The results showed that adverse events were 9–20 times more likely to be reported in the pharmacotherapy research papers than in the psychotherapy research papers, suggesting that pharmacotherapy research pays more attention to the possibility of adverse outcomes than psychotherapy research. In line with these findings, another study reviewed 132 randomized controlled trials of psychological interventions in patients for monitoring adverse events, side effects, and deterioration. Only 28 of the trials (21%) included information on adverse events, and of these, most had incomplete descriptions of how these adverse events were defined and monitored (Jonsson et al., 2014). While there has been some advancement, as indicated by a recent systematic review noting that 60% of published preregistered psychotherapy trials explicitly reported harmful events, there is still considerable heterogeneity in the conceptualization, monitoring, and reporting of these events (Klatte et al., 2023). This diversity impedes the accurate assessment of risks and benefits associated with psychotherapy interventions.

Part of the difficulty in investigating the adverse effects of psychotherapy is the lack of consensus on the most appropriate terms and definitions to describe these effects (Parry et al., 2016). One method often used by researchers to identify a potential negative response to psychotherapy is through standard patient outcome measures assessing symptom reduction (Whipple and Lambert, 2011). Recent evidence suggests that a non-trivial minority of patients experience a worsening of symptoms despite receiving psychological treatments that have been validated and are delivered appropriately. It has been estimated that between 5 and 10% of adult patients participating in clinical trials are worse off after treatment than they were before treatment began (Lambert, 2013), with slightly higher rates (7–15%) for patients with substance use disorders (Moos, 2005). However, it is difficult to determine how many of these patients would have deteriorated regardless of treatment (Berk and Parker, 2009). Perhaps some patients are already on a negative trajectory when they enter treatment, and this deteriorating course cannot be stopped. Others may experience unpleasant life events unrelated to treatment that lead to a worsening of symptoms (Lambert, 2013). Therefore, it cannot be assumed that deterioration on outcome measures *during* treatment means that this outcome was *caused* by the treatment. Furthermore, worsening of symptoms may not be the only type of negative effect of treatment. Even when treatment is beneficial, patients may still experience negative reactions, such as feeling emotionally overwhelmed, stigmatized, or impaired social functioning (e.g., strained family relationships). Thus, negative treatment effects are not synonymous with non-improvement or deterioration in treatment outcome measures. This is underscored by qualitative research on clients’ perspectives on their experiences in psychotherapy (e.g., Ladmanová et al., 2022; Vybíral et al., 2024). Clients’ psychotherapy experiences encompass a broad spectrum, including both helpful experiences (e.g., gaining a new perspective on themselves, feeling empowered, or feeling accepted by the therapist) and hindering experiences (e.g., feeling emotionally overwhelmed, feeling confused, or lack of guidance from the therapist). Curran et al. (2019) used task analysis, integrating qualitative research findings and client accounts, to develop a model of process factors that can potentially lead to negative or harmful effects in psychotherapy. Their findings emphasize the presence of potentially harmful factors at each stage of therapy that require attention. Both clients and therapists face challenges in addressing and identifying negative events in therapy. Clients frequently find it difficult to address negative experiences (e.g., Burton and Thériault, 2020; Calabro et al., 2024), contributing to the challenge of identifying them. Meanwhile, therapists may struggle to recognize negative events during therapy sessions and their own role in them (Hannan et al., 2005; Hatfield et al., 2010; Werbart et al., 2019; Dandachi-Fitz Gerald et al., 2022). If these negative events are not recognized, discussed, and satisfactorily resolved, they can have lasting negative consequences (e.g., a higher risk of poorer outcomes, less trust in therapy) (e.g., Burton and Thériault, 2020; Calabro et al., 2024), underscoring the importance of actively seeking feedback on clients’ positive and negative experiences as part of a collaborative therapeutic working alliance.

To date, however, there is no generally accepted definition or assessment method for negative effects of psychotherapy, although some proposals for a conceptual framework have been made (Berk and Parker, 2009; Linden, 2013; Parry et al., 2016). Linden (2013) proposed a framework for identifying and classifying adverse events in psychotherapy that follows a stepwise process. Adverse events are defined as all events that occur in parallel with psychotherapy, are patient-related, and have a negative quality. Such adverse events may be related or unrelated to the psychotherapy and include adverse changes in somatic or psychological symptoms, the patient’s sense of well-being, and social or occupational functioning. An example of a side effect (an adverse reaction to correct treatment) is increased anxiety during exposure therapy, the first-line treatment for phobias. The increase in anxiety that occurs when patients are exposed to cues related to the feared situation or sensation is considered a necessary part of the treatment, helping patients to overcome their fears through corrective learning. However, if there were another treatment that could achieve the same positive effect without burdening the patient with increased anxiety, that other treatment would be preferred. By this definition, side effects can be unexpected, but they can also be expected and sometimes even intended and unavoidable effects of treatment.

There are relatively few systematic studies investigating the occurrence and prevalence of negative effects of psychotherapy. To date, systematic research has been hampered by the lack of a clear definition of negative effects and the diversity of terms used, which has led to difficulties in developing adequate instruments to measure negative effects. In a review by Herzog et al. (2019), eight instruments for assessing negative effects of psychotherapy were systematically examined for their theoretical orientation and psychometric properties. The authors concluded that these instruments collectively cover a large number of domains, without reaching a consensus on which domains are the most important ones. They also found that the psychometric properties of a number of these instruments are insufficient. It has been suggested that the desired, positive effects of psychotherapy should also be included in assessment tools in order to minimize negative priming. An instrument with only items about negative experiences could potentially contribute to a nocebo effect due to negative expectations about the occurrence of side effects (Herzog et al., 2019; Moritz et al., 2019; but see Muschalla et al., 2023). Taking into account these findings on negative priming and the lack of a golden standard instrument for measuring negative reactions to psychotherapy, Dandachi-Fitz Gerald et al. (2024) developed a new instrument incorporating several points of improvement: the Positive and Negative Experiences of Psychotherapy Questionnaire (PNEP). This self-report questionnaire is based on the PANEPS (Peth et al., 2018; Moritz et al., 2019), the NEQ (Rozental et al., 2016), a literature review, and an evaluation of both the PANEPS and the NEQ by a panel of experiential experts. The panel emphasized the importance of including not only items concerning negative, but also positive experiences in order to be more representative of their own experiences with psychotherapy (Berghs, 2020). The PNEP consists of two subscales, negative and positive psychotherapy experiences, and a question asking respondents to rate how beneficial the therapy was. The term psychotherapy *experience* was chosen rather than psychotherapy *effect*, because the latter implies a causal relationship that is difficult to establish. In a sample of 200 participants, Dandachi-Fitz Gerald et al. (2024) found that 89.5% reported at least one negative treatment experience as measured by the PNEP. This percentage is close to the upper bound of the 22 to 93% range of negative treatment experiences found in various studies (e.g., Schneibel et al., 2017; Moritz et al., 2019; Rozental et al., 2019; Gerke et al., 2020).

Negative psychotherapy experiences clearly occur. However, the relationship between the occurrence of negative psychotherapy experiences and subsequent treatment outcome is still unclear. Negative experiences that occur during psychotherapy may be a transient phenomenon related to therapeutic interventions that are experienced as negative by the patient but are helpful in the long run. From this perspective, a temporary worsening of symptoms may sometimes even be necessary for improvement to occur. Change in psychotherapy is often nonlinear, with clients feeling worse before symptoms begin to decrease (Antichi and Giannini, 2023). Alternatively, such negative psychotherapy experiences may actually prevent patients from benefiting from therapy and lead to deterioration. In a pilot study, Brakemeier et al. (2018) examined the relationship between adverse events and treatment outcome in terms of response, remission, and relapse rates in a sample of 50 patients in a multidisciplinary inpatient setting for treatment-resistant chronically depressed patients. A self-report questionnaire specifically designed for use in inpatient settings was used to measure three categories of adverse events 6 to 12 months after discharge: subjective temporary worsening of depressive symptoms during treatment, interpersonal conflict with treatment staff or other patients, and major negative life changes after discharge. The results showed that only subjective temporary worsening of symptoms during treatment was associated with treatment outcome. That is, patients who reported a temporary worsening of symptoms were less likely to achieve remission. These results tentatively suggest that certain adverse events experienced by patients may have a negative impact on treatment outcome.

The current study aimed to examine the relationship between positive and negative psychotherapy experiences, as measured by the PNEP at mid-treatment, and treatment outcome, as measured by the OQ-45 in a sample of patients with mild to moderate mental health care problems receiving treatment in an ambulant setting. We hypothesized that (1) reporting of more positive experiences at mid-treatment would be associated with a more positive treatment outcome, whereas (2) reporting of more negative experiences would be associated with a less positive treatment outcome. For exploratory purposes, we were also interested in how positive and negative psychotherapy experiences at mid-and end-treatment would relate to OQ-45 at baseline and end-treatment, and to participants’ evaluation of how beneficial their treatment had been. We also explored whether there was a difference in psychotherapy experiences at mid-treatment between the group of patients that had clinically significantly improved on the OQ-45 at end-treatment, and those that did not. Finding a relationship between negative experiences and treatment outcome would suggest the importance of monitoring negative experiences during therapy in order to identify patients at risk of deriving less benefit from therapy and to intervene to alter their potential negative treatment trajectories. To our knowledge, this is the first study to examine the association between positive and negative psychotherapy experiences and treatment outcome.

## Methods

### Participants

**Figure 1:**
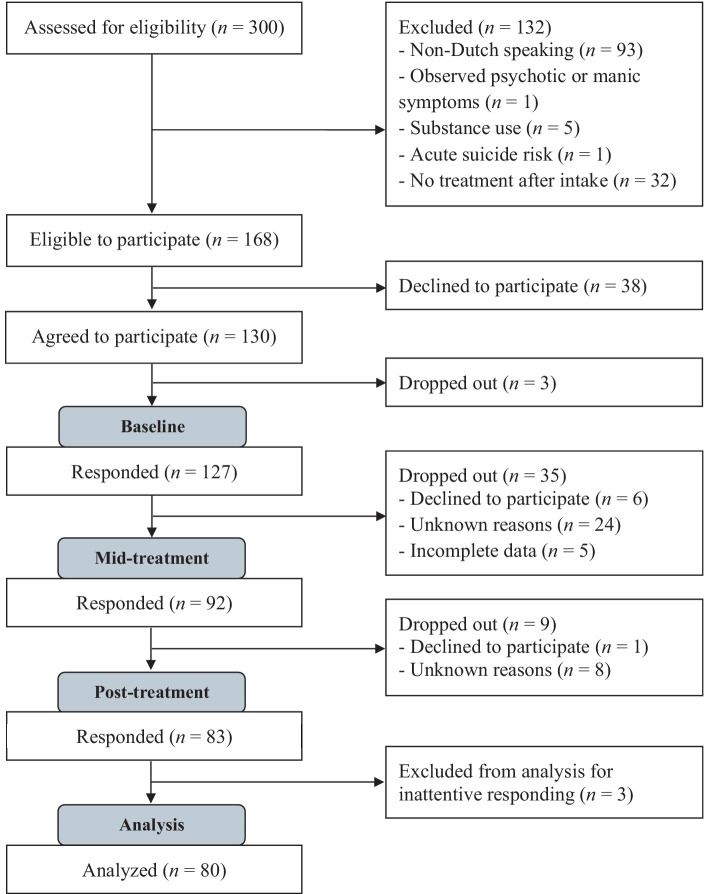
Flowchart of participant inclusion and exclusion.

Sample size was calculated *a priori* using G*Power (Faul et al., 2007). Prior effect size estimates were not available because the current study is the first to examine the relationship between positive and negative psychotherapy experiences and treatment outcome. For a credible range of effect sizes (with Cohen’s $f^{2}$ ranging from 0.08 to 0.15), power analysis indicated that the study would be powered at 80% (two-tailed, α = 0.05) with approximately 80 participants. Participants were patients aged 18–65 who were referred to an outpatient mental health service in Maastricht, the Netherlands, for treatment of a current DSM-5 disorder. The mental health care system in the Netherlands is divided into a so-called basic and specialized mental health care domain. The basic domain is intended for patients with a mental disorder of low to moderate severity, such as mild to moderate mood or anxiety disorders, or other disorders with only mild impairment in functioning and without suicidality, psychotic symptoms, or substance addiction, who can be helped with relatively short-term treatment with a maximum of 12 sessions. The specialized mental health care domain is intended for the treatment of more complex mental health problems and more severe psychiatric disorders, such as bipolar disorder or severe depression, (complex) posttraumatic stress disorder, and personality disorders, with a greater impairment in several areas of functioning and with no limit on the duration of treatment. For pragmatic purposes, patients referred to the basic mental health care domain were included in the study, as this domain has a high number of patient enrollment and high patient flow due to the short duration of treatment. Exclusion criteria were illiteracy, indication of intellectual disability, substance use disorder, observed psychotic or manic symptoms, and acute suicide risk, which are also the exclusion criteria for the basic mental health care domain in the Netherlands. Non-Dutch speaking patients were also excluded. Ethical approval was obtained from the standing Ethical Review Committee of the Faculty of Psychology and Neuroscience of Maastricht University [ERCPN 240_112_07_2021: v02]. A total of 300 patients were screened for eligibility. After screening, 168 patients were eligible to participate, of whom 130 (77.4%) agreed to participate in the study. Only complete data sets (baseline, mid-treatment, and end-treatment) were used for this analysis, resulting in a sample size of 83 participants. Three participants were subsequently excluded from the analysis due to inattentive responding, resulting in a final sample size of *N* = 80. See Figure 1 for a detailed flowchart of participants. Of note, initially, participants received only an email reminder to complete the questionnaires. After noticing a fairly high non-response rate and consequently study dropout, the researchers began contacting participants by phone to remind them to complete the questionnaires, which greatly improved the response rate. The sociodemographic characteristics of the sample analyzed (*N* = 80) are shown in Table 1. The mean age of the participants was 33.1 years (*SD* = 13.5), ranging from 18 to 69 years. The mean number of treatment sessions was 10 (*SD* = 3.8). As shown in Table 2, the mean OQ-45 total score at baseline was 72.1, which is within the clinical range, and decreased to 57.1 at the end of treatment. In total, 40 participants (50.0%) showed a clinically significant improvement on the OQ-45 from baseline to end-treatment, while 39 (48.8%) did not show a clinically significant change, and 1 participant (1.2%) significantly deteriorated on the OQ-45. Patients who dropped out of the study did not differ significantly from completers with respect to age [*t*(125) = 1.54, *p* = 0.127], gender [X2(1) = 0.22, *p* = 0.636], and baseline OQ-45 [*t*(122) = 0.56, *p* = 0.575].

**Table 1:** Sociodemographic characteristics of the sample (*N* = 80).

**Characteristic**	***n***	**%**
Gender		
Women	56	70.0
Men	24	30.0
Educational level		
Low	25	31.3
Medium	22	27.5
High	33	41.3
Relationship status		
In a relationship	36	45.0
Not in a relationship	44	55.0
Work status		
Employed	54	67.5
Unemployed	26	32.5
Primary diagnosis		
Depressive disorder	30	37.5
Anxiety disorder	28	35.0
Trauma-and stressor-related disorder	14	17.5
Personality disorder	3	3.8
Somatic symptom disorder	1	1.3
Obsessive-compulsive disorder	1	1.3
Eating disorder	1	1.3
Attention-deficit/hyperactivity disorder	1	1.3
Impulse control disorder	1	1.3

**Table 2:** Means, standard deviations, and correlations between variables.

	**Variable**	***M***	***SD***	**1**	**2**	**3**	**4**	**5**	**6**	**7**
1	OQ-45 baseline	72.10	19.78							
2	OQ-45 end-treatment	57.06	20.75	0.66**						
3	Positive experiences mid-treatment	18.19	7.40	−0.03	−0.30**					
4	Negative experiences mid-treatment	2.76	3.25	0.13	0.25*	−0.30**				
5	Positive experiences end-treatment	22.09	7.30	0.26*	−0.20	0.59**	−0.25*			
6	Negative experiences end-treatment	2.95	3.47	0.15	0.21	−0.27*	0.56**	−0.35**		
7	Overall evaluation mid-treatment	4.46	0.76	−0.08	−0.28*	0.73**	−0.29**	0.48**	−0.30**	
8	Overall evaluation end-treatment	4.72	1.08	0.20	0.03	0.18	−0.34**	0.52**	−0.35**	0.23*

*p < 0.05; **p < 0.01.

### Measures

#### Positive and negative experiences of psychotherapy questionnaire

The Positive and Negative Experiences of Psychotherapy Questionnaire (PNEP; Dandachi-Fitz Gerald et al., 2024) was used to measure the occurrence of positive and negative psychotherapy experiences at mid-and end-treatment assessments.

The PNEP consists of two subscales of items measuring both positive psychotherapy experiences (33 items) and negative psychotherapy experiences (36 items). Each PNEP item first provides respondents with a description of an experience (e.g., “I felt more confident about myself,” or, “I experienced more stress”) and asks them to indicate whether they experienced this. If participants respond affirmatively, they are asked to rate the intensity of the experience (5-point Likert scale: *not at all intense, to some extent, reasonably, very intense, and extremely intense*). Finally, participants are asked whether they attribute the experience entirely to the treatment they received, partly to the treatment and partly to other circumstances, or entirely to other circumstances. At the end of the PNEP, participants are asked to rate the extent to which they have benefited from their treatment (7-point Likert scale, *strongly deteriorated, deteriorated, slightly deteriorated, unchanged, slightly improved, improved and strongly improved*). First psychometric findings indicate that the PNEP is a sound instrument with adequate internal reliabilities (positive subscale: *α* = 0.94; negative subscale: *α* = 0.90) and quite good test–retest stability (positive subscale: *r* = 0.93; negative subscale *r* = 0.78) (Dandachi-Fitz Gerald et al., 2024). In the current study, Cronbach’s alpha values were 0.90 for the positive subscale and 0.81 for the negative subscale. For this study, a positive or negative experience was considered to be a treatment-related experience if (1) the item was answered affirmatively and (2) it was partly or entirely attributed to therapy. Total scores on both subscales were used to measure positive psychotherapy experiences (maximum total score of 33) and negative psychotherapy experiences (maximum total score of 36). In addition, the question asking participants to rate the extent to which they benefited from their treatment was used as a measure of subjective treatment evaluation.

#### Outcome questionnaire – 45 items

The outcome questionnaire – 45 items (OQ-45) (Lambert et al., 1996, 2004) is a self-report instrument designed for repeated measurement and consisting of 45 items, nine of which are reversed, asking respondents how they have felt in the past week (5-point Likert scale: *never, rarely, sometimes, frequently, almost always*). Higher scores reflect higher levels of dysfunction. The OQ-45 consists of three subscales designed to assess different domains of patient functioning: Symptomatic Distress, Interpersonal Relations, and Social Role. Internal consistency (Cronbach’s alpha) for the total score of the Dutch version ranges from 0.92 to 0.96 in university, community, patient, and community and patient combined samples. Test–retest reliability for the Dutch version was found to range from 0.79 to 0.82 for the total score (De Jong et al., 2008). In this study, the Cronbach’s alpha value was 0.94. For the current study, the total score was used as a measure of treatment outcome. A reliable change index (RCI) of 14 was used to determine clinically significant change from baseline to end of treatment.

#### Experimental check on data integrity

Five control items were added at the mid-and end-treatment assessments to check for inattentive responding (e.g., “If I read this item carefully, I will answer ‘Never’ here”) and overreporting of symptoms (e.g., “I experience such terrible headaches, that my feet hurt from it”) at mid-and end-treatment as a check on data integrity. Participants who failed on more than two of these items were excluded from the analysis.

### Design and procedure

The study used a longitudinal, prospective, correlational research design to examine the association between positive and negative effects of psychotherapy and treatment outcome. Participants were recruited during routine clinical interview sessions at the outpatient mental health care service. Recruitment occurred after the clinician assessed the eligibility for study participation. Participants were provided with a study brochure containing general information about the study and an informed consent form. They were informed that they could withdraw from the study at any time without any implications for their treatment. Diagnosis, psychotropic medications, and number of treatment sessions were obtained from patient records. Participants reported their age, gender, education level, work status, and relationship status. The PNEP was administered at mid-treatment (i.e., after four sessions) and at post-treatment. The OQ-45 was administered at baseline and post-treatment. Data on treatment duration (i.e., number of sessions) were collected at post-treatment. Supplementary Figure S1 visualizes the study steps.

### Transparency and openness

We report how we determined our sample size, all data exclusions, and all measures in the study, and we follow JARS (Appelbaum et al., 2018). All data and research materials are available at https://doi.org/10.34894/QZUNSW. Data were analyzed using IBM SPSS Statistics for Windows, Version 27.0. The study was preregistered on the Open Science Framework prospectively, before data were collected (view only link for peer review^1^). There are no previously published works or works that are currently in press or under review stemming from the same dataset as this manuscript.

### Data analysis

SPSS was used for all analyses. Data were checked for missing values and violation of assumptions (linearity, normality). Outliers were identified and examined. Study and treatment withdrawal was registered. Completers and non-completers were compared on baseline characteristics (i.e., demographics and OQ-45 at baseline). Descriptive statistics (means, standard deviations, percentages, and frequencies) were calculated to assess the percentages and levels of positive and negative treatment experiences and treatment outcome among the study participants. To test our hypotheses, a series of stepwise linear hierarchical regression analyses were conducted. First, a backward analysis was performed to identify potentially relevant covariates. Demographic variables, number of treatment sessions, treatment-unrelated positive and negative experiences at mid-treatment, and OQ-45 at baseline were entered as predictors of OQ-45 at end-treatment. Sequentially, non-significant predictors were removed. The criterion for removal was based on the significance level of the *F* value, which was set at 0.05. The remaining set of predictors was then used in a subsequent forward selection strategy. This forward regression analysis was performed in two steps. In the first step, the set of variables identified in the backward analysis was entered. In the next step, the two predictor variables of interest (i.e., total positive and negative psychotherapy experiences at mid-treatment) were added. For the exploratory analyses, we performed correlational analyses among variables, including PNEP positive and negative subscales at mid-and end-treatment, OQ-45 at baseline and end-treatment, and overall treatment evaluation at mid-and end-treatment. Additionally, we utilized independent samples t-test to compare the subgroup that exhibited clinically significant change on the OQ-45 from pre-to post treatment with the subgroup that did not show improvement.

## Results

### Positive and negative psychotherapy experiences at mid-treatment

All patients reported positive psychotherapy experiences at mid-treatment, ranging from 3 to 33 positive experiences, with a mean score of 18.2 (see Table 2). The most commonly reported experiences were related to the therapeutic relationship [e.g., feeling well informed (98.8%), understood (95.0%), and accepted (98.8%) by the therapist]. Positive experiences related to having more positive feelings, and to personal growth and acceptance were also frequently reported. The frequencies of all positive treatment experiences can be found in Supplementary Table S1.

At least one negative experience was reported by 55 (69%) of the participants. The mean number of negative experiences was 2.8, with a maximum of 13. The most commonly reported negative effects were related to experiencing more symptoms and emotional distress, such as feeling overwhelmed by emotions (35.0%), experiencing more negative thoughts and memories (26.3%), feeling more stress and/or tension (20.0%), and feeling vulnerable or unprotected (17.5%). Stigma-related experiences were also reported, such as feeling ashamed of having received treatment (10.0%), and fearing that others would find out that they had received treatment (7.5%). None of the participants reported experiencing verbal or sexual transgression by the therapist. The frequencies of all negative psychotherapy experiences can be found in Supplementary Table S2.

### Correlations between variables

As expected, baseline OQ-45 was significantly correlated with OQ-45 at end-treatment. Positive and negative psychotherapy experiences at mid-treatment were significantly correlated with positive and negative psychotherapy experiences at end-treatment, respectively. Reporting of more positive experiences at mid-treatment was significantly associated with a better outcome on the OQ-45 at the end of treatment. In contrast, negative experiences mid-treatment did not correlate with treatment outcome on the OQ-45. However, reporting more negative experiences was associated with reporting less positive experiences, both at mid-and end-treatment. Also, reporting more negative experiences at mid-treatment was associated with a less favorable overall treatment evaluation both at mid-treatment and end-treatment.

### Regression analysis

In a backward analysis, baseline OQ-45 and relationship status were identified as relevant predictors of OQ-45 at end-treatment. A forward stepwise linear regression analysis was then performed (see Table 3). No multicollinearity was found. In the first model, baseline OQ-45 and relationship status were entered, and in the second model, total positive and negative treatment experiences at mid-treatment were entered. Adding positive and negative treatment experiences significantly increased *R*^2^. The second model was found to be significant [*F*(4, 75) = 23.56, *p* < 0.001] and explained 56% of the variance in OQ-45 at end-treatment. Both baseline OQ-45 and relationship status significantly predicted OQ-45 at end-treatment. Being in a relationship at baseline was associated with lower OQ-45 scores at end-treatment. In addition, positive psychotherapy experiences at mid-treatment were a significant predictor of OQ-45 at end-treatment, even after controlling for the effects of baseline OQ-45 and relationship status, with more positive experiences associated with lower OQ-45 scores at end-treatment. The contribution of negative psychotherapy experiences at mid-treatment to the model was not significant.

**Table 3:** Hierarchical regression results for OQ-45 at end-treatment.

Variable	Model 1		Model 2	
	*β*	*SE*	*β*	*SE*
Constant		6.51		7.59
OQ-45 baseline	0.67***	0.09	0.65***	0.08
Relationship status	−0.24**	3.36	−0.18*	3.31
Positive experiences mid-treatment			−0.23**	0.23
Negative experiences mid-treatment			0.06	0.53
*R* ^2^			0.56	
Δ*R*^2^			0.06***	

*p < 0.05; **p < 0.01; ***p < 0.001.

### Comparison of psychotherapy experiences between improvers and non-improvers

We examined whether there was a difference in psychotherapy experiences at mid-treatment between the group of patients who showed clinically significant improvement on the OQ-45 at the end of treatment compared to baseline and those who did not. Participants with clinically significant improvement reported significantly more positive psychotherapy experiences at mid-treatment (*M* = 20.6, *SD* = 7.6) than participants without clinically significant improvement (*M* = 15.8, *SD* = 6.5); *t*(78) = 3.1, *p* = 0.003, *d* = 0.69. There was no difference in the amount of negative psychotherapy experiences at mid-treatment, *t*(78) = −0.9, *p* = 0.39, *d* = −0.19. No difference was found between these two groups in the types of experiences most commonly reported.

## Discussion

The aim of the present study was to examine the association between positive and negative psychotherapy experiences at mid-treatment and subsequent treatment outcome. First, all participants reported positive psychotherapy experiences at mid-treatment. At least one negative psychotherapy experience was reported by 55 (69%) of the participants at mid-treatment.

With regard to our two main hypotheses, the results can be summarized as follows. First, reporting more positive psychotherapy experiences at mid-treatment was associated with a more positive treatment outcome. Patients who reported more positive experiences at mid-treatment had a more favorable treatment outcome than patients who reported fewer of these positive experiences. The most commonly reported positive experiences were feeling accepted, understood, and supported by the therapist, and feeling that the treatment was well conducted, all of which are related to the therapeutic alliance (i.e., clarity and agreement about treatment goals and tasks, and the formation of a positive emotional bond, Bordin, 1994). A stronger therapeutic alliance has been consistently associated with better treatment outcomes across a range of psychotherapies (Flückiger et al., 2018), and a recent systematic review (Baier et al., 2020) of 37 psychotherapy studies identified the therapeutic alliance as a mediator of change or “treatment driver” that plays an important role in effective psychotherapy. Other commonly reported positive experiences in the present study were related to a better understanding of self, improved ability to deal with problems, and increased self-acceptance. These findings are consistent with previous research on what changes patients value in psychotherapy beyond symptom reduction (Binder et al., 2010; Hill et al., 2013; Timulak and Keogh, 2017), and suggest that such changes are positively associated with better treatment outcomes in terms of symptom improvement at the end of treatment.

Regarding the association between negative psychotherapy experiences and subsequent treatment outcome, our second hypothesis was not supported. That is, reporting more negative psychotherapy experiences did not predict a less favorable outcome after controlling for baseline psychological distress and relationship status. Nor was an inverse association found, which is consistent with previous research (Moritz et al., 2015) and provides further evidence against the “no pain, no gain” view that such negative experiences are necessary for patients to benefit from treatment. Although no association was found between negative experiences reported at mid-treatment and treatment outcome in terms of symptom reduction on the OQ-45, we did find that reporting more negative experiences was associated with reporting fewer positive experiences, and with a less positive overall evaluation of the treatment. These results are consistent with another recent study (Dandachi-Fitz Gerald et al., 2024) and suggest that negative experiences, while having no effect on symptomatic improvement, may be related to the way in which patients evaluate their treatment. These findings are similar to those reported for adverse side effects of pharmacological treatments, which also do not appear to have a direct impact on their mechanisms of action, but can negatively affect patients’ quality of life, treatment adherence (Dibonaventura et al., 2012; Franzoi et al., 2021; Fleming et al., 2022), and treatment satisfaction (Hughes et al., 2017). Although in the present study, negative psychotherapy experiences did not affect treatment outcome in terms of symptom reduction, it is important for patients to be informed about their potential occurrence so that they can make a better decision about whether to enter treatment (Blease et al., 2016). Crawford et al. (2016) conducted a large survey of patients receiving psychological treatment, asking respondents whether they had experienced lasting negative effects from treatment. Interestingly, respondents who said that they were not given enough information about the treatment before it started were more likely to report lasting bad effects. The authors suggest that properly informed patients may have more realistic expectations about the treatment, which may modulate their perception of negative effects. In addition, recent research suggests that monitoring and discussing side effects can improve the therapeutic alliance, which in turn may have a positive effect on treatment outcome (Muschalla et al., 2023).

In our study, the four most commonly reported negative experiences were feeling emotionally overwhelmed, having more negative thoughts and memories, an increase in stress and/or tension, and feeling vulnerable and unprotected. These findings align with the study by Dandachi-Fitz Gerald et al. (2024), who found that these four negative experiences were the most commonly reported in both a patient sample and a professional sample of psychologists who had received psychotherapy as part of their training. In the present study, both positive and negative psychotherapy experiences appeared to be relatively stable over time, with the reporting of more experiences at mid-treatment being associated with the reporting of more experiences at the end of treatment. Further research using follow-up assessments is needed to investigate whether these experiences persist after the end of treatment, or whether they represent a more transient phenomenon. It is important to note that clients’ subjective reporting of negative experiences does not invariably lead to worsened treatment outcome. Some negative experiences may even contribute positively to the therapeutic process. To aid clinicians in distinguishing between negative psychotherapy experiences that facilitate treatment progress and those that impede it, more research is warranted to elucidate the relationship between client-reported experiences and treatment outcomes. For now, considering that clients’ perspectives on their therapy experiences, particularly negative ones, are often overlooked, clinicians may utilize our findings to not only inform clients but also to systematically monitor both positive and negative experiences. By doing so, clinicians can better recognize early signs of client dissatisfaction or discomfort and respond timely.

### Strengths and limitations

The main strengths of this study were the use of a naturalistic setting, which allows the findings to be generalized to clinical practice, the use of a longitudinal prospective design, and the novelty of the research question. In addition, both positive and negative psychotherapy experiences were included, which is in accordance with what patients have been shown to find representative of their experience of psychotherapy (Dandachi-Fitz Gerald et al., 2024). Despite these strengths, the present study has several limitations. The PNEP is a self-report instrument and we cannot be sure how accurate people are in identifying positive and negative experiences and attributing them to a specific cause. In addition, diagnoses were not based on structured clinical interviews, such as the SCID-5, but on the therapists’ assessment following a clinical interview with the patient. Because the study used diagnosis only as a descriptive baseline characteristic, this assessment of diagnosis was considered to be acceptable. Another limitation is that patients were recruited from the basic mental health care domain. In general, patients referred to this segment have less severe mental health problems, making it difficult to generalize the present findings to other patient groups with more severe mental health problems. In addition, patients varied in terms of diagnosis and the type of treatment they received [e.g., cognitive behavioral therapy, eye movement desensitization and reprocessing (EMDR), acceptance and commitment therapy (ACT), and schema-focused therapy (SFT) interventions]. Due to the exploratory nature of the present study, this heterogeneity in diagnoses and treatment interventions is less of a concern. A future line of research might be to investigate whether specific positive and negative experiences are associated with different psychotherapies and/or with different diagnostic classifications. Another interesting line of future research might be to investigate the effects of informing both patients and therapists about the occurrence of negative psychotherapy experiences, and of monitoring for these experiences during treatment. A final limitation relates to the relatively high level of participant attrition between baseline and mid-treatment assessment, which increases the risk of attrition bias. Participants who dropped out of the study after baseline may have had more negative psychotherapy experiences or less positive treatment outcomes.

In conclusion, both positive and negative psychotherapy experiences are common and should not be overlooked. Importantly, in order for patients to provide informed consent to treatment, it is crucial that they are informed of the potential costs (i.e., treatment burden), risks, and benefits of that treatment (Blease et al., 2016; Dobler et al., 2018). Although positive experiences outnumber negative experiences, individuals referred for psychotherapy should be informed about the most common negative experiences that may also occur.

## Data availability statement

The datasets presented in this study can be found in online repositories. The names of the repository/repositories and accession number(s) can be found below: https://doi.org/10.34894/QZUNSW.

## Ethics statement

The studies involving humans were approved by Ethical Review Committee of the Faculty of Psychology and Neuroscience of Maastricht University. The studies were conducted in accordance with the local legislation and institutional requirements. The participants provided their written informed consent to participate in this study.

## Author contributions

RV: Conceptualization, Methodology, Writing – original draft, Writing – review & editing, Data curation, Formal analysis, Investigation, Project administration, Visualization. NB: Conceptualization, Methodology, Writing – review & editing, Formal analysis, Writing – original draft. BD-F: Methodology, Writing – review & editing, Conceptualization, Resources, Supervision, Writing – original draft.

## Footnotes

^1^ https://osf.io/jkvg3/?view_only=55de685342b84154acbab23ad951f9fd
