# Supplementary material for: No pain, no gain revisited: the impact of positive and negative psychotherapy experiences on treatment outcome
Source: Front Psychol. 2024 Jun 18;15:1378456. doi: 10.3389/fpsyg.2024.1378456 (PMC11220492; doi:10.3389/fpsyg.2024.1378456)
Supplement: Supplementary file 1 [file Table_1.DOCX]

**Supplementary Table 1**Frequency of Positive Psychotherapy Experiences at Mid-Treatment

| **PNEP item** | **Positive experience** | ***n*** | **%** |
| --- | --- | --- | --- |
| 18. | I thought the treatment was well executed. | 79 | 98.8 |
| 20. | The therapist informed me well about the treatment. | 79 | 98.8 |
| 24. | I felt accepted by the therapist. | 79 | 98.8 |
| 23. | The therapist was understanding and supportive. | 76 | 95.0 |
| 22. | I had a good relationship with the therapist. | 75 | 93.8 |
| 1. | I felt better. | 64 | 80.0 |
| 11. | I was proud to have tried the treatment. | 58 | 72.5 |
| 15. | I understood myself better. | 57 | 71.3 |
| 19. | I had confidence in the method of treatment. | 54 | 67.5 |
| 25. | I learned how to deal with problems better. | 54 | 67.5 |
| 8. | I learned to accept myself more. | 53 | 66.3 |
| 14. | I gained new hope. | 50 | 62.5 |
| 3. | I felt more comfortable in my own skin. | 48 | 60.0 |
| 2. | The symptoms for which I was seeking therapy diminished. | 46 | 57.5 |
| 5. | I felt calm and relaxed more often. | 46 | 57.5 |
| 12. | Others (family, partner, friends) were proud of me for being in therapy. | 46 | 57.5 |
| 4. | I could enjoy myself more. | 42 | 52.5 |
| 6. | I felt happy more often. | 40 | 50.0 |
| 26. | I learned where to go when I need help. | 39 | 48.8 |
| 28. | I gained more control over my life. | 39 | 48.8 |
| 9. | I gained more confidence in my own abilities. | 36 | 45.0 |
| 16. | I was able to grow as a person. | 35 | 43.8 |
| 29. | I learned to take more responsibility for myself. | 35 | 43.8 |
| 27. | I took better care of myself. | 31 | 38.8 |
| 13. | I learned to live more according to what I find valuable and important. | 29 | 36.3 |
| 30. | I was better able to carry out everyday activities. | 25 | 31.3 |
| 32. | Relationships with people close to me (partner, friends) improved. | 22 | 27.5 |
| 21. | The therapist prepared me well for the time after treatment. | 20 | 25.0 |
| 17. | I made plans for the future. | 19 | 23.8 |
| 31. | My relationship with my family improved. | 19 | 23.8 |
| 7. | I woke up feeling more fit and rested. | 16 | 20.0 |
| **PNEP item** | **Positive experience** | ***n*** | **%** |
| 10. | I had new, pleasant memories that I did not have before the treatment. | 15 | 18.8 |
| 33. | At my work/school/other daily activity, others knew about my therapy/diagnosis, and this had a positive effect on me. | 15 | 18.8 |

*Note. N* = 80.
