# Supplementary material for: No pain, no gain revisited: the impact of positive and negative psychotherapy experiences on treatment outcome
Source: Front Psychol. 2024 Jun 18;15:1378456. doi: 10.3389/fpsyg.2024.1378456 (PMC11220492; doi:10.3389/fpsyg.2024.1378456)
Supplement: Supplementary file 2 [file Table_2.DOCX]

**Supplementary Table 2**
Frequency of Negative Psychotherapy Experiences at Mid-Treatment

| **PNEP item** | **Negative experience** | ***n*** | **%** |
| --- | --- | --- | --- |
| 6. | I felt overwhelmed by emotions. | 28 | 35.0 |
| 3. | I had more negative thoughts and memories. | 21 | 26.3 |
| 1. | I felt more stress and/or tension. | 16 | 20.0 |
| 7. | I felt vulnerable or unprotected. | 14 | 17.5 |
| 2. | I had trouble sleeping. | 11 | 13.8 |
| 11. | I had new, unpleasant memories that I did not have before the treatment. | 11 | 13.8 |
| 16. | I did not feel any improvement. | 11 | 13.8 |
| 4. | I felt more anxious. | 10 | 12.5 |
| 9. | The symptoms for which I was seeking therapy worsened. | 10 | 12.5 |
| 12. | I was ashamed to have received psychological treatment. | 8 | 10.0 |
| 5. | I felt more depressed. | 7 | 8.8 |
| 23. | I did not understand the aim of the treatment well. | 7 | 8.8 |
| 15. | I am afraid that others will find out that I have received treatment. | 6 | 7.5 |
| 17. | I felt hopeless. | 5 | 6.3 |
| 18. | I have lost confidence in myself / my self-esteem has decreased. | 5 | 6.3 |
| 27. | I felt a distance in my relationship with the therapist. | 5 | 6.3 |
| 33. | I was unable to carry out daily activities (e.g. work, school, other activities). | 5 | 6.3 |
| 10. | New symptoms appeared that I did not have before I started treatment. | 4 | 5.0 |
| 14. | I felt that others thought I was crazy. | 4 | 5.0 |
| 19. | The treatment took longer than I had expected or was told. | 4 | 5.0 |
| 20. | I lost confidence in the treatment. | 4 | 5.0 |
| 24. | I did not think the therapist was skilled enough. | 4 | 5.0 |
| 28. | I did not have a good relationship with my therapist. | 4 | 5.0 |
| 22. | I felt I was given the wrong diagnosis. | 3 | 3.8 |
| 25. | I did not feel taken seriously by the therapist. | 3 | 3.8 |
| 26. | I did not feel understood by the therapist. | 3 | 3.8 |
| 8. | I had thoughts about how it would be better if I were not here, or about ending my life. | 2 | 2.5 |
| 29. | I felt too dependent on the therapist. | 2 | 2.5 |
| **PNEP item** | **Negative experience** | ***n*** | **%** |
| 21. | The treatment was only aimed at ‘getting rid of problems’; there was no positive goal we were trying to achieve. | 1 | 1.3 |
| 30. | I was afraid to make decisions without consulting the therapist first. | 1 | 1.3 |
| 32. | My relationship with my partner deteriorated. | 1 | 1.3 |
| 34. | Others at work/school/other daytime activities knew about my therapy/diagnosis, and this had a negative effect on me. | 1 | 1.3 |
| 13. | Others (family, partner, friends) were ashamed that I was receiving psychological treatment. | 0 | 0.0 |
| 31. | My relationship with my family deteriorated. | 0 | 0.0 |
| 35. | I experienced verbal violence, e.g. shouting/bashing/ mocking/coercion from the therapist. | 0 | 0.0 |
| 36. | I experienced sexual violence, e.g. sexually inappropriate comments, touching or sexual abuse by the therapist. | 0 | 0.0 |

*Note. N* = 80.
