# Supplementary figures and images for: No pain, no gain revisited: the impact of positive and negative psychotherapy experiences on treatment outcome
Source: Front Psychol. 2024 Jun 18;15:1378456. doi: 10.3389/fpsyg.2024.1378456 (PMC11220492; doi:10.3389/fpsyg.2024.1378456)

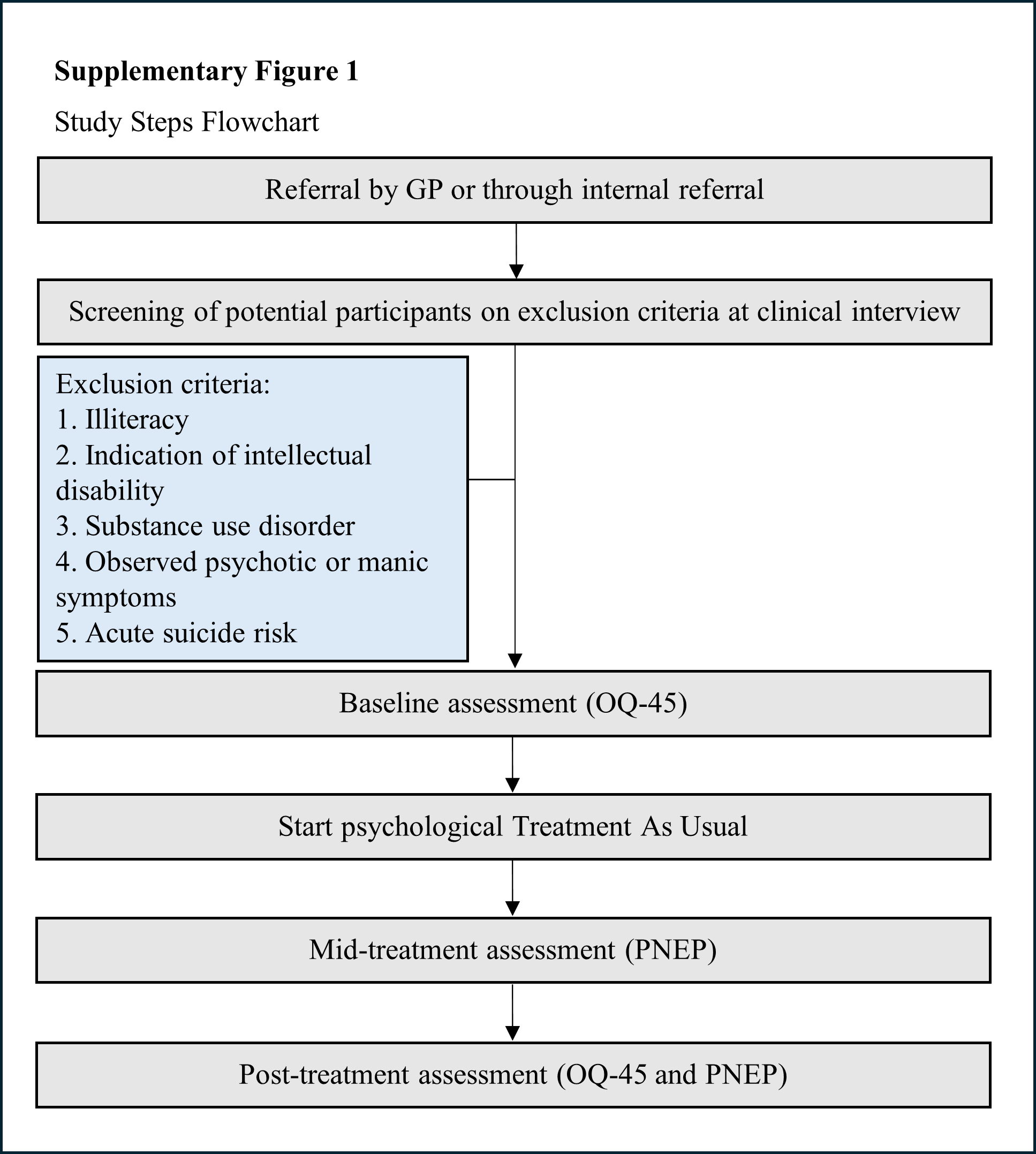

Supplement: Supplementary file 3 [file Image_1.TIF]
